# Unmasking a Rare Triad of Iron Deficiency Anemia, Esophageal Web, and Dysphagia in Plummer-Vinson Syndrome: A Case Report and Review of the Literature

**DOI:** 10.7759/cureus.116887

**Published:** 2026-09-25

**Authors:** Ramachandra Reddy, Karthik Rajaram Mohan, Saramma Mathew Fenn, Reethikarathan Rathanswamy Thiruneervannan, Kumar Appusamy

**Affiliations:** 1 Oral Medicine and Radiology, Vinayaka Mission's Sankarachariyar Dental College, Vinayaka Mission's Research Foundation (Deemed to be University), Salem, IND; 2 Oral Medicine and Radiology, Vinayaka Mission’s Sankarachariyar Dental College, Vinayaka Mission’s Research Foundation (Deemed to be University), Salem, IND

**Keywords:** barium esophagram, hematinic syrup, iron deficiency anemia, melanin pigmentation, plummer-vinson syndrome

## Abstract

Plummer-Vinson syndrome (PVS), also known as Paterson-Kelly syndrome or sideropenic dysphagia, is a rare disorder characterized by the triad of iron deficiency anemia, dysphagia, and esophageal webs. It predominantly affects middle-aged women and sometimes carries a risk of malignant transformation to esophageal and hypopharyngeal carcinoma, which makes early recognition clinically significant. This case report describes a 37-year-old woman who presented with a four-month history of a burning sensation and dysphagia to solid foods. Her physical examination revealed pale palpebral conjunctiva, and intraoral examination showed pale, pigmented buccal mucosa bilaterally, along with dental caries and multiple missing maxillary posterior teeth. Hematological investigations revealed severe microcytic hypochromic anemia with low serum ferritin, consistent with iron deficiency, and a barium swallow study demonstrated an esophageal stricture, confirming the diagnosis of PVS. The patient was advised to undergo prosthetic rehabilitation of her missing teeth to support adequate mastication and nutrient absorption, and was referred to a gastroenterologist for further management of the esophageal stricture and to a general physician for correction of iron deficiency anemia. This report highlights the clinical presentation, oral and hematological findings, diagnostic workup, and multidisciplinary management of PVS, and underscores the value of prompt barium swallow evaluation and gastroenterology referral in patients with iron deficiency anemia and dysphagia, given the syndrome's potential for malignant transformation.

## Introduction

Plummer-Vinson syndrome (PVS), or Paterson-Brown-Kelly syndrome, is a rare clinical entity characterized by iron-deficiency microcytic hypochromic anemia and dysphagia due to the presence of an esophageal web [1]. This syndrome is predominantly seen in middle-aged women, with a female predominance [2]. The association between anemia and dysphagia was first noted by Plummer in 1914, and later, in 1917, Mosher described the presence of esophageal webs in these patients [2]. In 1922, Vinson reported a series of 69 cases, which subsequently led to the syndrome being called PVS [3]. It was named after Mayo Clinic physicians Henry Stanley Plummer and Porter Paisley Vinson of the United States of America (USA) [3].

A similar condition was described by British laryngologists Donald Ross Paterson and Adam Brown-Kelly in 1919 [3]. Paterson suggested that the condition was precancerous and stated that the dysphagia in affected patients was caused by an esophageal web resulting in stricture. Brown-Kelly described the syndrome as comprising iron deficiency anemia, dysphagia, and an esophageal web [3].

## Case presentation

A 37-year-old woman reported to our department with a chief complaint of a burning sensation and dysphagia while eating solid foods for the past four months. Her past medical history revealed that she did not have diabetes. Her personal history revealed that she had no harmful oral habits, such as chewing betel or using smokeless tobacco. Her history revealed that the patient was anemic but was not on iron supplementation. Physical examination revealed pale palpebral conjunctiva Figure 1.

**Figure 1:**
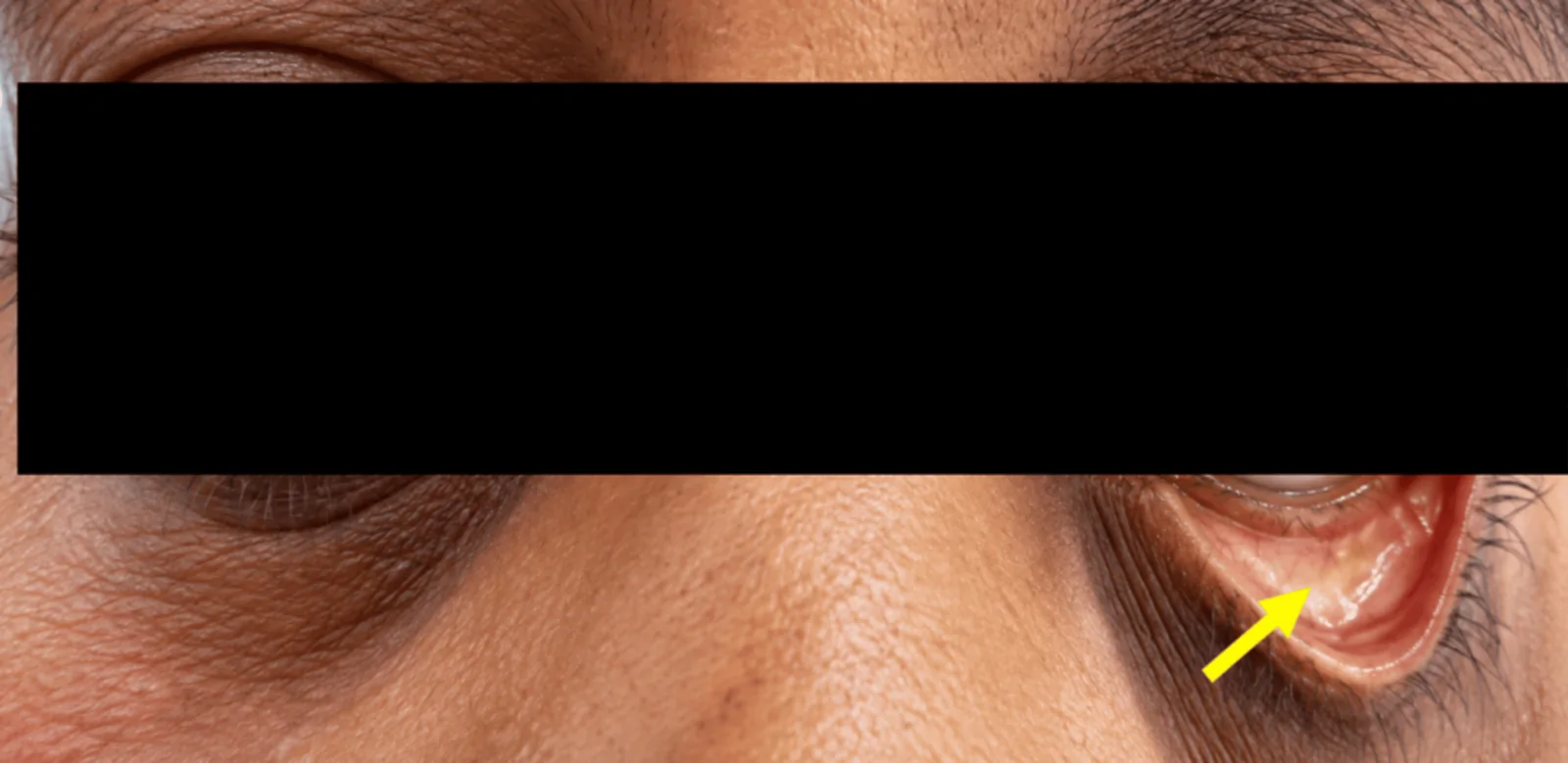
Extraoral examination. Pale palpebral conjunctiva (yellow arrow).

Intraoral examination revealed pale, pigmented right and left buccal mucosa; dental caries in the right maxillary first and second molars; and multiple missing and carious maxillary posterior teeth (Figures 2A-2C).

**Figure 2:**
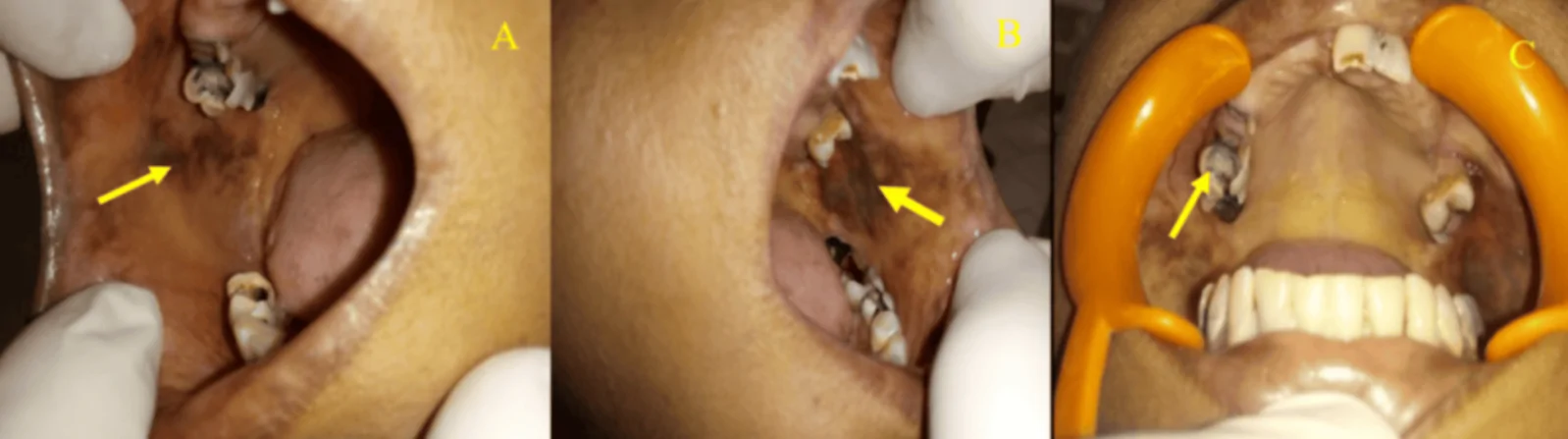
(A) Discrete greyish pigmentation on the right buccal mucosa. (B) Pigmentation on the left buccal mucosa. (C) Multiple missing and carious maxillary posterior teeth (yellow arrow).

The differential diagnosis for pallor of the oral mucosa includes iron deficiency anemia and oral submucous fibrosis. Oral submucous fibrosis occurs in patients with a betel nut chewing habit, which was absent in this case. The hematological laboratory parameters assessed are tabulated in Table 1.

**Table 1:** Hematological laboratory parameters.

Hematological laboratory parameters	Observed value	Normal value
Hemoglobin (g/dL)	7.4	12-15.5
Hematocrit	27.6%	36-44%
Mean corpuscular volume (MCV) (fL)	62.1	80-100
Red cell distribution width (RDW)	23.1%	12.1%-16.1%
Serum iron (µg/dL)	17	11-25
Ferritin (ng/mL)	3.1	13-150

Barium swallow study revealed an esophageal stricture (Figure 3).

**Figure 3:**
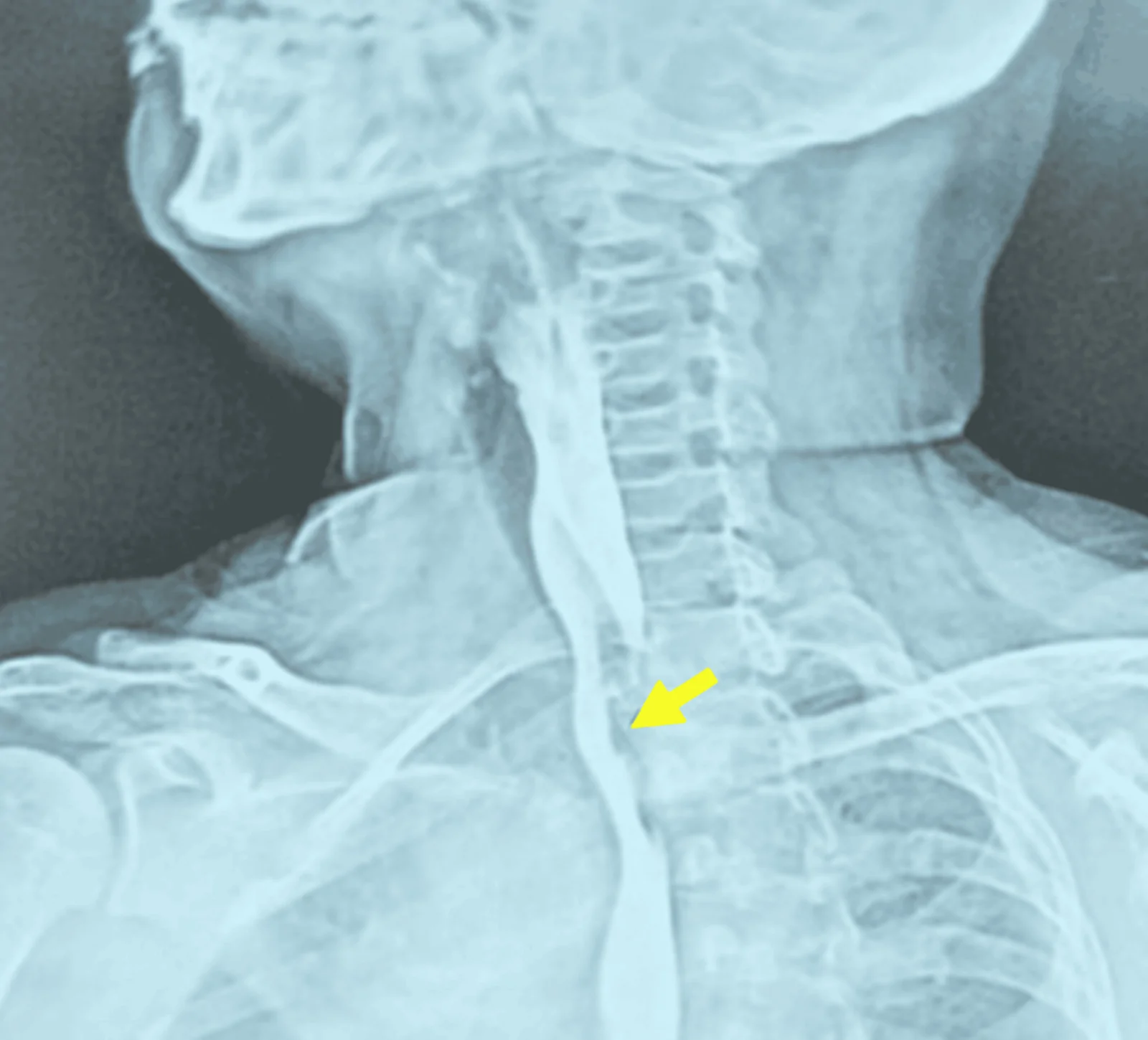
Barium swallow study revealed a characteristic "step-off appearance" suggestive of an esophageal web (yellow arrow).

Based on the clinical features and the radiological and hematological parameters, a final diagnosis of PVS was made. Oral hematinics were prescribed to correct the iron deficiency anemia, and the patient was referred to a gastroenterologist for evaluation and management of the esophageal stricture. As atrophic, dry oral mucosa associated with iron deficiency anemia predisposes to denture-related irritation and ulceration, oral hematinics were prescribed to correct the anemia, and prosthetic rehabilitation was planned to follow once the oral mucosa showed adequate improvement, thereby minimizing the risk of mucosal trauma from the prosthesis. Prosthetic rehabilitation of her missing teeth was advised concurrently to restore adequate masticatory function, thereby supporting nutrient intake and complementing the medical management of her anemia.

The clinical significance of this case report lies in the early identification of an esophageal stricture by barium swallow and prompt referral to a gastroenterologist, which aids in early screening of the esophagus to rule out esophageal and pharyngeal carcinoma - a known complication of PVS - thereby reducing mortality and improving quality of life in these patients.

## Discussion

PVS is a rare disease that presents with iron deficiency anemia and esophageal webs resulting in dysphagia, initially of solid foods and later of liquids. This condition is associated with an increased incidence of esophageal and hypopharyngeal cancer [1]. A retrospective, decade-long, single-center study of 132 confirmed cases of PVS reported a mean patient age of 43.5 years (range, 16-76 years), with 85.6% of patients being female - findings that closely mirror the demographic profile of the present case [1]. In that cohort, single-session Savary-Gilliard bougie dilation successfully relieved dysphagia in the majority of patients, and the overall risk of upper gastrointestinal malignancy was 4.5%, underscoring the importance of endoscopic surveillance in patients with PVS [1].

PVS presents with the classic triad of iron deficiency anemia, dysphagia, and esophageal webs [2]. PVS is more frequent in females than in males, with a female-to-male ratio of 8:1 [2]. PVS is defined by upper esophageal webs, post-cricoid dysphagia, and iron deficiency [2,3]. People with PVS are more likely to develop fibrosis of the oral mucosa, which results in pain during mastication and speech [2]. Consequently, increased plaque accumulation in such patients raises the incidence of periodontal disease, dental caries, and poor oral hygiene [2]. PVS primarily affects women aged 30-60 years [3]. The clinical signs and symptoms of iron deficiency anemia include pallor, tiredness or weakness, tachycardia, and fatigue [3]. Other features of PVS include esophagitis, cardiospasm, achlorhydria, nail abnormalities such as koilonychia or clubbing, splenomegaly, goiter, dermatitis, seborrhea, hyperkeratosis, conjunctivitis, visual disturbances, keratitis, and blepharitis [3].

The diagnosis of PVS is based on a combination of clinical history, laboratory investigations, and radiographic studies. Laboratory tests typically reveal microcytic hypochromic anemia, and endoscopic examination of the esophagus can confirm the presence of a web or stricture. Management of PVS primarily involves addressing the underlying iron deficiency anemia via administration of oral or intravenous iron supplements. In some cases, dysphagia may improve with iron supplementation alone. However, in cases where dysphagia persists, the esophageal web may require mechanical dilation or surgical intervention. A literature review of cases of PVS is summarized in Table 2.

**Table 2:** Literature review of cases of Plummer-Vinson syndrome PVS: Plummer-Vinson syndrome

Author	Year	Age/gender	Clinical description
Vohra et al. [1]	2024	1-year-old/male	Iron supplements did not resolve dysphagia, and esophageal dilation is needed to open the blocked enteral pathway.
Kale et al. [2]	2023	-	Iron deficiency anemia can have many detrimental health consequences.
Kakumani et al. [3]	2024	53-year-old/female	PVS is defined by dysphagia, iron deficiency anemia, and esophageal webs.
Alfaris et al. [4]	2023	16-year-old/female	PVS is associated with dysphagia of gradual onset, starting with solid foods and progressing to liquids over years.
Jessamy et al. [5]	2023	39-year-old/female	Endoscopic-guided glucocorticosteroid injection of triamcinolone acetonide into the esophageal mucosa benefited refractory benign esophageal stenosis in PVS.
Mohan et al. [6]	2022	-	Esophageal squamous cell carcinoma occurred in 6.2% of PVS cases.
Harmouch et al. [7]	2021	88-year-old/female	A food bolus trapped in the cervical esophageal region required removal via esophagogastroduodenoscopy.
Obeidat et al. [8]	2021	29-year-old/female	PVS, the triad of dysphagia, iron deficiency anemia, and esophageal webs, is a relatively rare disease entity.
Patel et al. [9]	2021	63-year-old/female	Video-fluoroscopic swallow study revealed esophageal-and pharyngeal-phase dysphagia with food entrapment.
Goh et al. [10]	2020	46-year-old/female	Painless, gradually progressive dysphagia involving solids.
Radochová et al. [11]	2020	65-year-old/female	The incidence of PVS is less than 1% among the population with iron deficiency anemia.
Gade et al. [12]	2019	39-year-old/female	PVS is precancerous with high malignant potential.

## Conclusions

PVS, also known as Paterson-Kelly syndrome or sideropenic dysphagia, is a rare medical condition that is characterized by the triad of iron deficiency anemia, dysphagia, and esophageal webs resulting in an esophageal stricture. Whenever iron deficiency anemia is encountered in a patient with dysphagia, a barium swallow study or fluoroscopic visualization of the esophageal mucosa is essential to rule out an esophageal web or stricture near the upper esophageal sphincter.

Early diagnosis and prompt referral to a gastroenterologist are necessary to screen for life-threatening post-cricoid carcinoma, which can occur in patients with PVS. Additionally, prosthetic rehabilitation of missing teeth should be considered to support adequate mastication and nutrition.
